# Health Care Providers’ Knowledge of HPV Vaccination, Barriers, and Strategies in a State With Low HPV Vaccine Receipt: Mixed-Methods Study

**DOI:** 10.2196/cancer.7345

**Published:** 2017-08-11

**Authors:** Echo L Warner, Qian Ding, Lisa Pappas, Julia Bodson, Brynn Fowler, Ryan Mooney, Anne C Kirchhoff, Deanna Kepka

**Affiliations:** ^1^ Cancer Control and Population Sciences Department Huntsman Cancer Institute Salt Lake City, UT United States; ^2^ College of Nursing University of Utah Salt Lake City, UT United States; ^3^ Study Design and Biostatistics Center School of Medicine University of Utah Salt Lake City, UT United States; ^4^ Biostatistics Core Huntsman Cancer Institute Salt Lake City, UT United States; ^5^ Department of Pediatrics University of Utah Salt Lake City, UT United States

**Keywords:** health care provider, human papillomavirus, human papillomavirus vaccine, mixed methods, knowledge

## Abstract

**Background:**

Human papillomavirus (HPV) vaccination is below national goals in the United States. Health care providers are at the forefront of improving vaccination in the United States, given their close interactions with patients and parents.

**Objective:**

The objective of this study was to assess the associations between demographic and practice characteristics of the health care providers with the knowledge of HPV vaccination and HPV vaccine guidelines. Furthermore, our aim was to contextualize the providers’ perceptions of barriers to HPV vaccination and strategies for improving vaccination in a state with low HPV vaccine receipt.

**Methods:**

In this mixed-methods study, participating providers (N=254) were recruited from statewide pediatric, family medicine, and nursing organizations in Utah. Participants completed a Web-based survey of demographics, practice characteristics, HPV vaccine knowledge (≤10 correct vs 11-12 correct answers), and knowledge of HPV vaccine guidelines (correct vs incorrect). Demographic and practice characteristics were compared using chi-square and Fisher exact tests for HPV knowledge outcomes. Four open-ended questions pertaining to the barriers and strategies for improving HPV vaccination were content analyzed.

**Results:**

Family practice providers (52.2%, 71/136; *P*=.001), institutional or university clinics (54.0%, 20/37; *P*=.001), and busier clinics seeing 20 to 29 patients per day (50.0%, 28/56; *P*=.04) had the highest proportion of respondents with high HPV vaccination knowledge. Older providers aged 40 to 49 years (85.1%, 57/67; *P*=.04) and those who were a Vaccines for Children provider (78.7%, 133/169; *P*=.03) had the highest proportion of respondents with high knowledge of HPV vaccine recommendations. Providers perceived the lack of parental education to be the main barrier to HPV vaccination. They endorsed stronger, consistent, and more direct provider recommendations for HPV vaccination delivered to parents through printed materials available in clinical settings and public health campaigns. Hesitancy to recommend the HPV vaccine to patients persisted among some providers.

**Conclusions:**

Providers require support to eliminate barriers to recommending HPV vaccination in clinical settings. Additionally, providers endorsed the need for parental educational materials and instructions on framing HPV vaccination as a priority cancer prevention mechanism for all adolescents.

## Introduction

In 2013, the US President’s Cancer Panel identified provider recommendations as one of three priorities for improving the rates of human papillomavirus (HPV) vaccination [1]. A strong provider recommendation of the HPV vaccine reflects up to a 5-fold increase in the decision by parents to vaccinate their child [2]. Multiple national organizations have echoed their support for providers to deliver strong recommendations for the HPV vaccine to eligible adolescents, including the American Academy of Family Physicians, the American Academy of Pediatrics, the American College of Obstetricians and Gynecologists, the American College of Physicians, the Centers for Disease Control and Prevention (CDC), and the Immunization Action Coalition [3]. Research on strategies to improve the consistency and quality of provider recommendations is pivotal to achieving the Healthy People 2020 goal of 80% HPV vaccination coverage set by the CDC [4].

Knowledge about HPV vaccines influences the providers’ intention to recommend HPV vaccination to their patients [5-7]. Low knowledge of the benefits of HPV vaccination among providers may contribute to low HPV vaccination rates in regions such as the Intermountain West, inclusive of Utah, Idaho, Nevada, Wyoming, Colorado, Montana, Arizona, and New Mexico [8]. In 2015, Utah was ranked the 49th state for HPV vaccine initiation among females (47.8%) and among the lowest for males (40.9%) aged 13 to 17 years [9]. Although knowledge deficits about HPV vaccines and HPV vaccine guidelines among the providers in Utah have not been described, previous research indicates that there is a high prevalence of missed opportunities for HPV vaccination in Utah [10]. Missed opportunities may reflect providers’ misconceptions or lack of knowledge about HPV vaccination. In addition, contextual factors such as cultural or religious assumptions regarding adolescents’ sexual practices may influence providers’ perceptions of HPV and their subsequent recommendation of the vaccine to their patients [11,12]. Thus, improving provider recommendations of the HPV vaccine to their patients first requires assessments of providers’ knowledge about HPV vaccines and HPV vaccine guidelines.

Theoretically informed approaches to improving HPV vaccination are necessary to advance research and practice in this area. The social ecological framework (SEF) is a health promotion model that encompasses multiple levels of influence. In the SEF, individual, interpersonal, and organizational characteristics constitute three of the five levels of influence on a public health intervention. Multilevel targeted interventions promote healthy practices such as the administration of HPV vaccines to prevent HPV-related morbidity and mortality [13]. For example, individual, interpersonal, and organizational SEF levels are represented in this study as parent and patient, health care provider, and organizational characteristics, respectively. By examining these characteristics from the health care providers’ perspectives, the SEF provides the theoretical foundation for understanding how these characteristics influence providers’ readiness to deliver a strong recommendation for HPV vaccination to patients and parents.

Moreover, the exposure of health care providers to the health care system, parents, and patients gives them unique perspectives on the clinical barriers and strategies for improving HPV vaccination. In this mixed-methods study, we describe providers’ knowledge of HPV vaccines and HPV vaccine guidelines and their perceptions of barriers to and strategies for improving HPV vaccination in Utah, which is a state with low HPV vaccination rates. We aimed to assess associations of demographic and practice characteristics with providers’ knowledge of HPV vaccination and HPV vaccination guidelines to identify provider groups with knowledge deficits. Providers’ perceptions of the barriers to and strategies for improving HPV vaccination were described to contextualize the results.

## Methods

Mixed-method approaches that combine qualitative and quantitative data resources provide a more complete description of a phenomenon than a single methodological approach alone [14,15]. Using a Web-based survey, our goal was to identify demographic and practice characteristics that are associated with providers’ knowledge about HPV vaccination and HPV vaccination guidelines in a state with a low HPV vaccination rate. Qualitative open-ended survey questions were used to further contextualize the findings from the survey analysis by describing providers’ perceptions of barriers to and strategies for improving HPV vaccination. The usability and technical functionality of the survey were assessed during pilot testing before data collection occurred. This study was deemed exempt research by the institutional review board of the University of Utah.

### Participants and Data Collection

During three periods from 2014-2015, a self-administered closed survey was distributed via email listservs to 3 statewide provider organizations, with sample sizes of approximately 600, 740, and 330 for pediatrics, family medicine, and nursing, respectively. The survey comprised 58 items, with 1 to 4 questions per page. Participants received notification of a forthcoming opportunity to participate in a research study, with the option to opt out from further contact (n=1). Eligible participants who did not opt out received an additional email invitation to complete the Web-based survey within 2 weeks. Two biweekly reminder emails were then sent within 4 weeks after the initial email. Anonymous submission of the completed survey constituted consent. Participants had the option to receive a US $20 Amazon gift card or make a US $20 donation to a local children’s hospital. The approximate response rates were as follows: pediatrics 18.0% (108/600), family practice 21.8% (161/740), and nurse practitioners 39.1% (129/330). Of these, 65 participants were excluded because they were not a pediatrician, family medicine physician, or nurse practitioner (eg, office staff and medical assistant), and 79 participants were excluded because they did not see patients in a clinical setting. The final sample of 254 participants who were analyzed comprised 75 pediatricians, 136 family medicine physicians, and 43 nurse practitioners.

### Independent Variables

Demographics included age, sex, race, marital status, and religion. Practice characteristics included practice location, Vaccines for Children (VFC) provider status, specialty type, practice type, practice size, number of patients per day, number of patients per week, most common form of patient payment, and provider-reported majority Hispanic population. Variable selection was guided by the SEF and included factors that represented multiple levels of influence, including individual, interpersonal, and community (eg, parents, patients, health care providers, organizations, and public policy). Variable selection was also based on extant literature and our previous research in Utah related to HPV vaccination.

### Outcome Measures

On the basis of a review of the literature, two HPV knowledge measures were measured (see Table 1): knowledge of HPV vaccination and knowledge of HPV vaccination guidelines. Knowledge of HPV vaccination was measured for each participant based on their responses to 12 true or false questions resulting in a score ranging between 0 and 12. This cutoff selection was based on the distribution of the data along a natural median divide. For analysis, HPV vaccination knowledge scores summarized into a binary variable with ≤10 indicating low knowledge and 11 to 12 indicating high knowledge.

The second outcome, knowledge of HPV vaccination guidelines, was measured for each respondent based on 3 questions about the timing and age of HPV vaccination. For analysis, we aggregated responses into a binary variable, with those who incorrectly answered any of the 3 questions as lower knowledge and those who answered all 3 questions correctly as high knowledge.

### Statistical Analysis

Summary statistics were reported for demographic and practice characteristics. Statistics were calculated for nonmissing data as indicated in Tables 2-5. Chi-square and Fisher exact tests were used for examining associations in univariate analyses with Stata version 14.1 (StatCorp LP). All *P* values were two-sided and considered significant at *P*=.05.

### Qualitative Data and Analyses

Qualitative data were extracted from 4 open-ended questions of the Web-based survey to describe providers’ perceptions of barriers to and strategies for improving HPV vaccination among males and females to “ground” the quantitative results. Grounding is a mixed-methods technique for combining qualitative and quantitative data to contextualize a phenomenon [14,15]. Responses were read and reread by 2 authors to familiarize with the data and identify themes. A deductive coding structure was created using levels of the SEF and revised as coding developed. Themes pertaining to providers’ perceptions at interpersonal (parents, patients, and providers) and organizational (health care system or public policy) levels of the SEF are described herein. Pertinent differences in providers’ perceptions about HPV vaccination for girls and boys are described.

**Table 1:** Outcome variable questions and responses.

Question	Correct response	Knowledge outcome
Vaccine leads to long-lasting immunity.	True	HPV^a^vaccination
Vaccine does not cause adverse side effects.	True	HPV vaccination
Vaccine protects against genital warts in addition to cervical cancer.	True	HPV vaccination
Condom use in patients does not decrease after vaccination.	True	HPV vaccination
Offering vaccination provides an opportunity to discuss sexuality issues with patients.	True	HPV vaccination
The likelihood of patients having sex does not increase after vaccination.	True	HPV vaccination
HPV vaccination is highly effective at preventing cervical cancer precursors.	True	HPV vaccination
Almost all cervical cancers are caused by HPV infection.	True	HPV vaccination
Women who have been diagnosed with HPV should not be given HPV vaccine.	False	HPV vaccination
The incidence of HPV in women is highest among women in their 30s.	False	HPV vaccination
Genital warts are caused by the same HPV types that cause cervical cancer.	False	HPV vaccination
A pregnancy test should be performed prior to giving HPV vaccine.	False	HPV vaccination
When is HPV vaccination recommended?	Before the beginning of sexual activity	HPV vaccine guideline
The recommended age for HPV vaccination in adolescent girls is?	Subjects aged 11-12 years	HPV vaccine guideline
The recommended age for HPV vaccination in adolescent boys is?	Subjects aged 11-12 years	HPV vaccine guideline

^a^HPV: human papillomavirus.

## Results

### Demographic and Practice Characteristics Associated With HPV Vaccination Knowledge and Guidelines

Participants included 136 family practice physicians, 75 pediatricians, and 43 nurse practitioners. No demographic factors were associated with providers’ knowledge of HPV vaccination (see Table 2).

In Table 3, specialty was associated with knowledge; family practice physicians had the highest proportion of providers with high HPV vaccination knowledge (52.2%, 71/136), whereas pediatricians had the lowest (26.7%, 20/75 *P*=.001). Providers from institutional or university settings (54.0%, 20/37) and primary care or other (50.5%, 49/97) had higher proportions of high HPV knowledge than private care (35.7%, 30/84) and hospital or urgent care clinics (15.6%, 5/32; *P*=.001). Providers who saw ≥15 patients per day had a higher proportion of high HPV knowledge (15-19 patients: 47.8%, 33/69; 20-29 patients: 50.0%, 28/56; ≥30 patients: 44.7%, 20/50) than providers who saw <15 patients per day (27.8%, 20/72; *P*=.04).

**Table 2:** Univariate analysis of demographic characteristics associated with human papillomavirus vaccination knowledge (N=254).

Demographics		Human papilloma virus vaccination knowledge		*P* value
		Lower knowledge (N=148)	High knowledge (N=106)	
		n (%)	n (%)	
**Age, in years**				.46^a^
	18-29	14 (56.0)	11 (44.0)	
	30-39	47 (52.2)	43 (47.5)	
	40-49	43 (64.2)	24 (35.8)	
	≥50	44 (61.1)	28 (38.9)	
**Sex**^b^				.57^a^
	Male	76 (59.8)	51 (40.2)	
	Female	71 (56.3)	55 (43.7)	
**Race**^b^				.21^a^
	White	134 (57.0)	101 (43.0)	
	Other^c^	13 (72.2)	5 (27.8)	
**Marital status**				.87^a^
	Single, divorced, widowed	22 (59.5)	15 (40.5)	
	Married, living as married	126 (58.1)	91 (41.9)	
**Religion**				.84^a^
	Latter-day Saint	70 (56.9)	53 (43.1)	
	Other religion	47 (61.0)	30 (39.0)	
	No religion	31 (57.4)	23 (42.6)	
**Location**^b^				.11^a^
	Salt Lake, Utah, or Davis counties	131 (60.1)	87 (39.9)	
	Other counties	16 (45.7)	19 (54.3)	

^a^Chi-square test.

^b^Missing values: Sex=1; Race=1; Location=1.

^c^Other includes black or African American, American Indian or Alaska Native, Asian, Native Hawaiian or Pacific Islander, Other.

**Table 3:** Univariate analysis of practice characteristics associated with human papillomavirus vaccination knowledge (N=254).

Practice characteristics		Human papillomavirus vaccination knowledge		*P* value
		Lower knowledge (N=148)	High knowledge (N=106)	
		n (%)	n (%)	
**Vaccines for children provider status**^a^				.06^b^
	Yes	94 (55.6)	75 (44.4)	
	No or Do not know	44 (60.3)	29 (39.7)	
	Do not provide vaccines^c^	10 (90.9)	1 (9.1)	
**Specialty**				*.001*^d^
	Pediatrician	55 (73.3)	20 (26.7)	
	Family practice physician	65 (47.8)	71 (52.2)	
	Nurse practitioner	28 (65.1)	15 (34.9)	
**Practice type**^a^				*.001*^d^
	Private (solo or group)	54 (64.3)	30 (35.7)	
	Primary care or Other^e^	48 (49.5)	49 (50.5)	
	Institutional or University settings	17 (46.0)	20 (54.0)	
	Hospital or Urgent care clinic	27 (84.4)	5 (15.6)	
**Practice size (number of physicians)**^a^				.36^d^
	1-5	47 (54.0)	40 (46.0)	
	6-10	37 (66.1)	19 (33.9)	
	>10	60 (57.7)	44 (42.3)	
**Number of patients per day**^a^				*.04*^d^
	<15	52 (72.2)	20 (27.8)	
	15-19	36 (52.2)	33 (47.8)	
	20-29	28 (50.0)	28 (50.0)	
	≥30	30 (60.0)	20 (40.0)	
**Number of patients per week**^a^				.09^d^
	<25	29 (74.4)	10 (25.6)	
	25-49	53 (55.8)	42 (44.2)	
	≥50	63 (55.3)	51 (44.7)	
**Most common patient payment**^a^				.31^d^
	Private insurance	86 (54.4)	72 (45.6)	
	Medicaid or Children's Health Insurance Program	35 (64.8)	19 (35.2)	
	Uninsured, Self-pay, Other, or Do not know	26 (63.4)	15 (36.6)	
**Patient population is Hispanic majority**				.59^d^
	Yes	15 (53.6)	13 (46.4)	
	No	133 (58.9)	93 (41.1)	

^a^Vaccines for children provider not applicable or missing=1; Practice type not applicable or missing=4; Practice size not applicable or missing=7; Number of patients per day other, not applicable, or missing=7; Number of patients per week other, not applicable, missing=6; Most common patient payment not applicable or missing=1.

^b^Fisher exact test.

^c^Individuals who see patients but do not provide vaccinations (eg, oncology).

^d^Chi-square test. Italics indicate *P* value less than .05.

^e^Includes ambulatory care, primary care clinic, health department, federally qualified health center, and other.

Tables 4 and 5 indicate that a lower proportion of providers aged 30 to 39 years (65.6%, 59/90) correctly identified HPV vaccination guidelines than those in other age groups (18-29 years: 80.0%; 20/25, 40-49 years: 85.1%, 57/67; ≥50 years: 75.0%, 54/72; *P*=.04). More VFC providers (78.7%, 133/169) correctly identified HPV vaccination recommendations compared with other providers (*P*=.03).

**Table 4:** Univariate analysis for demographic characteristics associated with human papillomavirus vaccine recommendation knowledge (N=254).

Demographics		Human papillomavirus vaccine recommendation knowledge		*P* value
		Lower knowledge (N=64)	High knowledge (N=190)	
		n (%)	n (%)	
**Age, in years**				*.04*^a^
	18-29	5 (20.0)	20 (80.0)	
	30-39	31 (34.4)	59 (65.6)	
	40-49	10 (14.9)	57 (85.1)	
	≥50	18 (25.0)	54 (75.0)	
**Sex**^b^				.26^a^
	Male	36 (28.3)	91 (71.7)	
	Female	28 (22.2)	98 (77.8)	
**Race**^b^				.05^a^
	White	56 (23.8)	179 (76.2)	
	Other^c^	8 (44.4)	10 (55.6)	
**Marital status**				.27^a^
	Single, divorced, widowed	12 (32.4)	25 (67.6)	
	Married, living as married	52 (24.0)	165 (76.0)	
**Religion**				.82^a^
	Latter-day Saint	29 (23.6)	94 (76.4)	
	Other religion	20 (26.0)	57 (74.0)	
	No religion	15 (27.8)	39 (72.2)	
**Location**^b^				.72^a^
	Salt Lake, Utah, or Davis counties	56 (25.7)	162 (74.3)	
	Other counties	8 (22.9)	27 (77.1)	

^a^Chi-square test. Italics indicate *P* value less than .05.

^b^Missing values: Sex=1, Race=1, and Location=1.

^c^Other includes black or African American, American Indian or Alaska Native, Asian, Native Hawaiian or Pacific Islander, Other.

**Table 5:** Univariate analysis for practice characteristics associated with human papillomavirus vaccine recommendation knowledge (N=254).

Characteristics		Human papillomavirus vaccine recommendation knowledge		*P* value
		Lower knowledge (N=64)	High knowledge (N=190)	
		n (%)	n (%)	
**Vaccines for children provider status**^a^				*.03*^b^
	Yes	36 (21.3)	133 (78.7)	
	No or Do not know	22 (30.1)	51 (69.9)	
	Do not provide vaccines^c^	6 (54.6)	5 (45.4)	
**Specialty**				.20^d^
	Pediatrician	15 (20.0)	60 (80.0)	
	Family practice physician	34 (25.0)	102 (75.0)	
	Nurse practitioner	15 (34.9)	28 (65.1)	
**Practice type**^a^				.72^d^
	Private (solo or group)	22 (26.2)	62 (73.8)	
	Primary care or Other^e^	21 (21.7)	76 (78.3)	
	Institutional or University settings	9 (24.3)	28 (75.7)	
	Hospital or Urgent care clinic	10 (31.2)	22 (68.8)	
**Practice size (number of physicians)**^a^				.07^d^
	1-5	26 (29.9)	61 (70.1)	
	6-10	17 (30.4)	39 (69.6)	
	>10	18 (17.3)	86 (82.7)	
**Number of patients per day**^a^				.20^d^
	<15	19 (26.4)	53 (73.6)	
	15-19	21 (30.4)	48 (69.6)	
	20-29	16 (28.6)	40 (71.4)	
	≥30	7 (14.0)	43 (86.0)	
**Number of patients per week**^a^				.35^d^
	<25	11 (28.2)	28 (71.8)	
	25-49	28 (29.5)	67 (70.5)	
	≥50	24 (21.0)	90 (79.0)	
**Most common patient payment**^a^				.07^d^
	Private insurance	32 (20.2)	126 (79.8)	
	Medicaid or Children's Health Insurance Program	16 (29.6)	38 (70.4)	
	Uninsured, Self-pay, Other, or Do not know	15 (36.6)	26 (63.4)	
**Patient population is Hispanic majority**				.98^d^
	Yes	7 (25.0)	21 (75.0)	
	No	57 (25.2)	169 (74.8)	

^a^Vaccines for children provider not applicable or missing=1; Practice type not applicable or missing=4; Practice size not applicable or missing=7; Number of patients per day other, not applicable, or missing=7; Number of patients per week other, not applicable, or missing=6; Most common patient payment not applicable or missing=1.

^b^Fisher exact test. Italics indicate *P* value less than .05.

^c^Individuals who see patients but do not provide vaccinations (eg, oncology).

^d^Chi-square test.

^e^Includes ambulatory care, primary care clinic, health department, federally qualified health center, and other.

The following results describe health care providers’ perceptions of barriers to HPV vaccination and strategies for improving HPV vaccination with accompanying illustrative quotes presented in the text and in Table 6. Each section is separated by (1) individual, (2) interpersonal, and (3) organizational constructs of the SEF, including (1) parents and patients, (2) health care providers, and (3) organizations, respectively. There were 74.4% (189/254) participants who responded to at least one of the 4 open-ended questions and 48.4% (123/254) participants who responded to all 4 questions.

### Providers’ Perceptions of HPV Vaccination Barriers

#### Barriers Related to Parents and Patients

In the open-ended questions, providers described concerns about sexual activity and promiscuity (n=69), vaccine refusal or reluctance (n=62), inadequate or incorrect parental knowledge (n=96), and low perceived risk of HPV (n=67) as the most common barriers to vaccination for parents and patients (see Table 6). To providers, parents’ perceptions about their child’s sexual activity influenced their decisions about HPV vaccination. One provider observed:

I do see a lot of moms “explain” the vaccine to their children saying, “It would be a good idea in case you were raped” rather than in case you had a sexual partner with HPV.

Providers responded that parents believed that the HPV vaccine increases sexual promiscuity, is unnecessary because their child is not sexually active, and that their child is not at risk for HPV infection. Providers connected parents’ concerns about sexuality with perceived risk of HPV infection. For example, one provider stated:

...if they’ve remained virginal, they assume the partner they marry is virginal and thus they aren’t at risk [for HPV]. Not thinking their partner might not be truthful OR that this marriage might not last and they could be exposed when they remarry, which by then [they] could be past immunization age.

Providers listed inadequate or incorrect parental knowledge as a barrier to vaccination about the purpose of HPV vaccination (Table 6). Providers felt that parental “misconceptions” were the result of parents being “very misinformed by relatives, or friends.” For example, a common endorsed barrier to vaccinating boys was the perception that HPV vaccines only prevent cervical cancer.

#### Barriers Related to Health Care Providers

Only a few respondents identified providers’ barriers to HPV vaccination. However, there were some concerns such as vaccination not being a priority (n=19). One provider stated:

We occasionally forget the vaccine at sick visits.

Some providers were openly unsupportive of HPV vaccination (n=16). One provider stated:

Without a history of homosexuality, I do not see a great advantage to the immunization of boys.

Whereas some providers felt HPV vaccines were not cost-effective, others expressed skepticism, stating they wanted “more science showing benefit in men” (n=13). A provider downplayed the need for HPV vaccination by stating:

Issues of sexually transmitted disease do not seem to be an issue in my clinical setting.

#### Barriers Related to Organizations

Organizational barriers to HPV vaccination included cost (n=32), completing follow-up doses (n=22), and infrequency of vaccinating at regular well-child or primary care visits (n=16).

### Perceptions of HPV Vaccination Improvement Strategies

#### HPV Vaccination Improvement Strategies for Parents and Patients

Parental education was the most common strategy for improving HPV vaccination (n=81). Providers felt that education should focus on reducing negative sexual connotations about the HPV vaccine. One provider relayed:

Basically, debunking the myth that it leads to more sex.

Providers felt that parents could be educated directly during clinic visits and through broader community health promotion campaigns. Informing parents about the prevalence of HPV within their community was suggested. One provider stated:

Better understanding that it is a ubiquitous virus and infects nearly everyone in the world, regardless of sexual partner number.

In some instances, providers’ perceptions varied by gender, with different ideas for vaccinating girls and boys (n=23, Table 6). Providers felt that parents need information about the efficacy of the HPV vaccine for reducing HPV-related morbidity among males.

#### HPV Vaccination Improvement Strategies for Providers

The most common suggestion for improving HPV vaccination by the providers was to tailor recommendations (n=23) and to focus on preventing cancer rather than sexually transmitted infections (n=18). Providers also felt that routine HPV vaccination would reduce parental and patient hesitancy (n=17, Table 6). Providers indicated that vaccine hesitancy was related to low perceived risk among parents and patients. Therefore, providers emphasized the importance of framing HPV vaccination recommendations:

...discussing the fact that [patients] can be exposed from a future husband who did not know he was infected.

Another provider echoed this perception:

Emphasizing that nonsexual intercourse exposure results in HPV acquisition and that there are respiratory and oral cancers associated too.

**Table 6:** Thematic findings and examples by levels of the social ecological framework (SEF).

Main theme and SEF^a^level		Subtheme	Sample quotes
**Perception of vaccine barriers**			
	Parents and Patients	Sexual activity and promiscuity (n=69)	“Their parents’ opinions regarding the teen’s sexuality [obviate the] legitimacy of the vaccine.”
		Vaccine refusal or reluctance (n=62)	“For some reason it is okay for women to have PAP exams but it is scandalous to get the vaccine that can prevent the cancer Pap exams detect.”
		Inadequate or incorrect parental knowledge (n=96)	“...very misinformed by relatives, or friends.”
		Low perceived risk of human papillomavirus (HPV) infection (n=67)	“They underestimate the risks of not being vaccinated. And overestimate the risks of vaccination.”
	Providers	Vaccine not a priority (n=19)	“We occasionally forget the vaccine at sick visits.”
		Not supportive of HPV vaccine (n=16)	“...[HPV vaccination] is a commercial success for HPV vaccines manufacturers; however, cervical cancer is not a pandemic disease and could be better controlled under personal choices than other diseases that [patients] must be vaccinated against.” “I live in a community where most teenagers are not sexually active until they get...It is hard to recommend a series of 3 somewhat painful shots to teenagers who are not planning to be sexually active until they get married.”
		More scientific evidence desired (n=13)	“...more science showing benefit in men.”
	Organizational	Cost (n=32)	“I recommend HPV in those that participate in VFC, but once they are 19 and older, it is too expensive.” “I’m a big proponent of vaccines, but the cost-benefit analysis of HPV just doesn’t support its widespread use. $400 is way too expensive…The HPV vaccines don’t obviate the need for pap smears, so what are we gaining here? Nothing.” “Make it free. Otherwise, I don’t have any plans to recommend it.”
		Completing follow-up doses (n=22)	“If it were not a series, they forget to finish it.” “Infrequent preventive visits. Difficulty completing the series.”
		Infrequency of visits (n=16)	“[There are] not enough well child visits to get in the entire series.”
**Perceptions of vaccine improvement strategies**			
	Parents and Patients	Education (n=81)	“Discussion about rates of infection in Utah especially in suburban areas and discussion about cervical cancer and its causes as a television campaign.”
		Gender differences (n=23)	“Better information about genital warts, anal cancer and other diseases caused by HPV that affect boys, and can be minimized by use of the vaccine.”
	Providers	Cancer prevention focus (n=18)	“Focusing on cancer prevention ‘later in life’ is more effective—especially when the discussion can be combined with the discussion about meningococcal meningitis and tetanus/pertussis. [HPV vaccination] is just a routine part of the preteen triad of immunizations.”
		Make HPV vaccination routine practice (n=17)	“To make it more routine like it is expected to get it in medical culture rather than this optional/additional vaccine.”
		Tailored recommendation (n=23)	“Discussing the fact that [patients] can be exposed from a future husband who did not know he was infected.”
		Educational information (n=22)	“I need some information sheets, reassurance sheets, on side effects and safety, which are easy to hand out.”
	Organizational	Public policy and standing orders (n=22)	“Adding it to the list of required vaccines for junior high and high school.”

^a^SEF: social ecological framework.

Providers endorsed the need for better educational information to be displayed in health clinics and comprehensible educational information on HPV vaccination to share with parents (n=22, Table 6).

#### HPV Vaccination Improvement Strategies for Organizations and Policy

Providers expressed support for public policy requiring HPV vaccination for school enrollment (n=22, Table 6). One provider also felt that standing orders for HPV vaccination would improve consistency in HPV vaccination.

## Discussion

### Principal Findings

This study is the first to describe providers’ knowledge of HPV vaccination and HPV vaccination guidelines, with added context of providers’ perceptions related to the barriers to and facilitators of HPV vaccination. Despite Utah’s very low HPV vaccination prevalence, another study with providers in Utah using similar survey items to assess providers’ knowledge of HPV indicated a substantially lower proportion of providers with correct knowledge compared with our sample (mean proportion of correct responses=57.7% vs 79.4%; [16]). Yet provider endorsement of HPV vaccination varies. There are some significant correlates of lower vaccination knowledge with provider demographics, which are described hereafter to inform future efforts targeted toward providers’ recommendation of HPV vaccination In addition, our qualitative results provide essential context for improving provider recommendations in states with low HPV vaccination. This study makes an important contribution to existing literature by using a mixed-methods design to describe providers’ perceptions of vaccine barriers that suppress HPV vaccination in a state with low HPV vaccine receipt.

Examination of multiple levels of the SEF is integral to designing effective HPV vaccination interventions. On an individual and interpersonal level, health care practice characteristics that were associated with lower knowledge of HPV vaccination and guidelines among providers in Utah include provider specialty (eg, pediatricians and nurse practitioners), practice type (eg, private practice and hospitals or urgent care clinics), and number of patients seen per day (eg, <15 and ≥30 patients per day). Additionally, younger providers (aged 30-39 years) and older providers (aged ≥50 years) had lower knowledge compared with those who were middle aged (40-49 years). The lower level of HPV vaccination knowledge among providers aged 30 to 39 years warrants attention. Given that HPV vaccination may not have been approved at the time of their clinical training, it is possible that these individuals may not have received training on HPV vaccination as a part of their clinical curriculum. Moreover, as new clinicians, these providers may have yet to establish robust continuing education opportunities to learn about HPV vaccines and guidelines. Thus, targeted opportunities for continuing education for those who have completed their medical or nursing training within the last 10 to 15 years may be merited. Continuing education for more established providers may help improve knowledge about HPV vaccination.

Providers who saw adolescent patients but did not routinely provide vaccinations, as well as those who were not VFC providers had lower knowledge about HPV vaccination and guidelines than did VFC providers. One explanation for this finding may be that VFC providers are potentially more accustomed to routinely providing HPV vaccines and thus may be more knowledgeable about this vaccine. In addition, the differential distribution of clinicians by specialty, with more family medicine providers than physicians practicing in rural areas [17], may have influenced our results on providers’ knowledge of HPV vaccination and guidelines. Although we did not examine the influence of rurality in this study, prior research has documented deficits in patient-provider communication about HPV vaccines from parents in rural areas as compared with those in urban areas [18].

Despite finding several associations between provider demographics and knowledge, the most compelling finding from this study was from our qualitative analyses demonstrating providers’ overwhelming perception of an immediate need for improved parental education regarding HPV vaccines. Misinformation among parents was portrayed by providers as the strongest and most consistent barrier to vaccination. Providers described how parental beliefs regarding sexuality and HPV vaccination impede HPV vaccination and make it difficult to deliver a strong recommendation in support of HPV vaccination. Providers expressed frustration at not having access to educational materials that they need to accurately and efficiently communicate with parents and patients about HPV vaccination. However, improvements in parental knowledge alone may not eliminate hesitancy toward HPV vaccination [19]. Continued promotion of HPV vaccination on an individual, interpersonal, organizational, and community level is needed to support providers’ strong recommendations for HPV vaccination to 11- to 12-year-old adolescents in Utah. Providers also endorsed public health campaigns as a strategy to inform parents of the ubiquity of HPV infection in their community by relaying local data on HPV prevalence for both males and females. In addition, providers supported framing the HPV vaccine as a cancer prevention mechanism for males and females. Lastly, providers felt that state policies requiring HPV vaccination would be the most powerful way to improve HPV vaccination. The feasibility of these strategies should be further explored.

Although health care providers’ hesitancy was not explicitly noted as a barrier to HPV vaccination, our qualitative analysis revealed that some providers have persistent negative perceptions of HPV vaccination. This reticence to endorse HPV vaccination has not only been observed in Utah but has also been described in national surveys [20]. Whereas parents report variation in the quality of provider recommendations, those who receive a strong endorsement for HPV vaccination are much more likely to choose vaccination [21]. Providers’ hesitancy to discuss sexual health, lack of time to address parental concerns about vaccine efficacy and safety, and perceptions of low self-efficacy to guide parents’ decisions about vaccination may discourage strong recommendations for HPV vaccination [12,22,23]. Furthermore, the lack of parental knowledge about HPV vaccination, which is noted as a barrier by providers, may be an unintended consequence of providers’ low knowledge about HPV vaccination and guidelines, potentially creating a situation in which providers with lower knowledge avoid discussing the HPV vaccine with their patients. Given the powerful impact of the strength and quality of providers’ HPV vaccine recommendations on parents’ decisions to vaccinate [21], providers in these settings may indeed benefit from education on the costs and benefits of HPV vaccination and the consequences of an unvaccinated population. Future research is warranted that explores the association between providers’ knowledge about HPV vaccination as well as guidelines and the administration of the HPV vaccine.

### Limitations

Limitations of this study include sampling of providers across a single state, which could be a potential threat to external validity. However, our results may be generalizable to other states with a low HPV vaccination rate and to states in the Intermountain West region. This depiction of HPV vaccination in Utah may be incomplete because we neither investigated perceptions of parents, patients, and communities nor the policies that influence HPV vaccination in Utah. Only 48.4% of providers responded to all 4 open-ended questions, thus nonresponse bias may exist in the qualitative findings, which means that those who did not respond to the open-ended questions may hold different perspectives on HPV vaccine barriers and strategies with regard to HPV vaccination for girls and boys. Our response rate was low, which may indicate that the knowledge of HPV vaccination and guidelines among providers who chose not to participate may differ. The variation in, and overall low response rate, among the different provider groups may have introduced differential bias to the results. Additionally, given the changing nature of listserv membership, it is possible that some providers may not have had equal opportunities to participate in the survey if they were added or removed from the listserv during the data collection period. However, we have no reason to believe that knowledge and perceptions of HPV vaccination would have been different for those who were migrating into and out of the sample for this reason.

HPV vaccination knowledge is commonly operationalized using a variety of measurement tools and survey items. Whereas standardized tools have been developed for measuring parental knowledge, tools that measure health care providers’ knowledge have yet to be tested. Utilization of standardized measurement tools to assess HPV vaccination knowledge among health care providers may facilitate comparisons across future studies. Lastly, we did not ask providers to report the exact location of their health care practice, which limited the data analyses.

### Conclusions

Utah’s vaccination rates are among the lowest in the United States. Theoretically informed interventions to improve vaccination through provider recommendations need to fully appreciate the public health benefit of HPV vaccination. This study provides evidence that provider-based HPV vaccine interventions must extend beyond improving providers’ knowledge about vaccination. Our analysis revealed that providers have knowledge of HPV vaccination and guidelines, but contextual factors accentuate the need for supporting providers in administering strong, consistent, and high-quality recommendations for the HPV vaccine in Utah. Recognizing the importance of provider’s experiences, we summarized their suggestions for improving HPV vaccination and recommend that providers’ perspectives be considered in the development of future interventions. Specifically, providers consider parental misconceptions to be the strongest barrier to HPV vaccination in Utah. Yet, they believe that misinformation can be corrected through direct parental education and broad public health campaigns. Providers’ recognize the value parents place on the dissemination of accurate information through clinical settings and appreciate the importance of a strong provider recommendation. In summary, providers in Utah have high knowledge about HPV vaccination, but they need support in correcting misinformation that persists at multiple levels of the SEF, including among patients, parents, colleagues, and communities.

## Abbreviations

CDC: Centers for Disease Control and Prevention
HPV: human papillomavirus
SEF: social ecological framework
VFC: Vaccines for Children
